# Internal and External Resources of the Lifestyle Leaders

**DOI:** 10.1093/geroni/igaa057.2073

**Published:** 2020-12-16

**Authors:** Martina Raue, Lisa D’Ambrosio, Taylor Patskanick, John Rudnik, Adam Felts, Julie Miller

**Affiliations:** 1 Massachusetts Institute of Technology, Cambridge, Massachusetts, United States; 2 MIT, Cambridge, Massachusetts, United States; 3 MIT AgeLab, Cambridge, Massachusetts, United States

## Abstract

With older age, people experience declines in resources and face new challenges. The goal of this study was to understand how resource decline affects the oldest olds’ well-being, but also to learn who they trust and where they go for advice in areas such as health, finances, and technology. This sample of 30 participants between the ages of 85 and 95 was generally resource-rich, scoring highest on self-esteem and optimism and lowest on mastery. Self-esteem and optimism correlated with financial resources, indicating a significant role of finances in this rather wealthy sample. Well-being was predicted by self-esteem and physical health. Presumably, their high levels of self-esteem compensate for the loss of other resources among the oldest old. The majority of lifestyle leaders trust in other people, and while friends and family are very important sources of advice, searching online was equally often mentioned as a source when looking for advice.

